# Modulation of T Cell Metabolism and Function through Calcium Signaling

**DOI:** 10.3389/fimmu.2013.00324

**Published:** 2013-10-11

**Authors:** Kelley M. Fracchia, Christine Y. Pai, Craig M. Walsh

**Affiliations:** ^1^Department of Molecular Biology and Biochemistry, The Institute for Immunology, University of California Irvine, Irvine, CA, USA

**Keywords:** calcium, metabolism, T cells, immune system, CRAC, reactive oxygen, DRAK2

## Abstract

As a vital second messenger in the activation of lymphocytes, the divalent cation Ca^2+^ plays numerous roles in adaptive immune responses. Importantly, Ca^2+^ signaling is essential for T cell activation, tolerance of self-antigens, and homeostasis. Supporting the essential role of Ca^2+^ signaling in T cell biology, the Ca^2+^ regulated protein phosphatase calcineurin is a key target of pharmacologic inhibition for preventing allograft rejection and for autoimmune therapy. Recent studies have highlighted the unique role of Stim1 and Orai1/2 proteins in the regulation of store-operated/calcium release activated calcium (CRAC) channels in the context of T cells. While Ca^2+^ is known to modulate T cell activation via effects on calcineurin and its target, nuclear factor of activated T cells (NFAT), this second messenger also regulates other pathways, including protein kinase C, calmodulin kinases, and cytoskeletal proteins. Ca^2+^ also modulates the unique metabolic changes that occur during in distinct T cell stages and subsets. Herein, we discuss the means by which Ca^2+^ mobilization modulates cellular metabolism following T cell receptor ligation. Further, we highlight the crosstalk between mitochondrial metabolism, reactive oxygen species (ROS) generation, and CRAC channel activity. As a target of mitochondrial ROS and Ca^2+^ regulation, we describe the involvement of the serine/threonine kinase DRAK2 in the context of these processes. Given the important roles for Ca^2+^ dependent signaling and cellular metabolism in adaptive immune responses, the crosstalk between these pathways is likely to be important for the regulation of T cell activation, tolerance, and homeostasis.

## Introduction

The adaptive immune system exhibits a wide potential for responding to infectious agents encountered throughout a lifetime. Notably, T cells play a critical role in mounting an appropriate response through the activation of their T cell receptor (TCR) upon the recognition of specific antigen presented through the major histocompatibility complex (MHC). This foreign peptide: MHC complex activates a T cell with specificity for the foreign antigen, culminating in proliferation, and differentiation into effector cells. In order to stimulate naïve T cells, ligation of the TCR by antigen must be accompanied by a costimulatory signal. Costimulation serves as a mechanism to modulate the strength of the TCR signal and promote higher gene expression of immunomodulatory cytokines like interleukin-2, as well as other factors that facilitate T cell proliferation and differentiation (1, 2). When a naïve T cell becomes activated, in the context of proper costimulation, it executes developmental reprograming characterized by rapid growth, proliferation, and acquisition of specialized effector functions. Given that this is an energetically demanding process, T cells undergo biochemical and biophysical reprograming to meet these requirements (3).

A key mediator of T cell signaling is the divalent cation calcium (Ca^2+^). Indeed, this second messenger is so important to T cell biology that its downstream signaling serves as a vital pharmacological target for the treatment of autoimmune disease and the prevention of chronic allograft rejection (4). As with many cell types, ligation of surface molecules that induce phospholipase C leads to mobilization of intracellular calcium, with depletion of Ca^2+^ from the endoplasmic reticulum (ER). However, it has long been known that for sustained signaling downstream of Ca^2+^ release, T cells bear special plasma membrane Ca^2+^ channels that promote the influx of extracellular Ca^2+^ (5). While beyond the scope of the present review, recent work in several laboratories has revealed that T cells and a select few other cell types, express plasma membrane channels that are activated in response to depletion of intracellular stores of Ca^2+^ (6). These calcium release activated calcium (CRAC) channels are activated by Stim1, an ER membrane protein that itself is induced upon depletion of Ca^2+^ stores in ER. Major efforts are currently underway to understand the biophysics of CRAC activation, and importantly, to determine if manipulation of CRAC signaling may be of therapeutic benefit.

In the present review, we examine the link between cellular metabolism and calcium signaling in the context of T cells. As described, Ca^2+^ modulates a variety of signaling cascades downstream of the TCR and costimulatory receptors, many of which impinge upon bioenergetic and biosynthetic pathways needed for T cell clonal expansion, differentiation, and homeostasis. First, we briefly outline several important control points that orchestrate cellular metabolic changes following TCR stimulation. Next, we discuss the crosstalk between mitochondrial metabolism and CRAC activity, describing the influence of the metabolic status of a given T cell on Ca^2+^ mobilization and signaling. Finally, the impact of the release of mitochondrial reactive oxygen species (ROS) on Ca^2+^ signaling is considered, with a focus on the immunoregulatory serine/threonine kinase DRAK2 in this process.

## Modulation of Cellular Metabolism Following T Cell Activation

Although one might consider metabolic regulation a mundane housekeeping function of cells, it is becoming apparent that specific metabolic programs are induced in distinct T cell subsets and developmental stages. For example, the metabolic status of naïve T cells is significantly different from their activated counterparts. Naïve T cells actively maintain a quiescent state through engagement of both intracellular signaling pathways and cell extrinsic signals (7), resulting in efficient use of available energy sources (8). These quiescent lymphocytes maintain a catabolic state and do not actively take up nutrients, nor do they secrete effector cytokines (9). Once committed to a T cell fate in the thymus, cellular metabolism plays a vital role in the development and proliferation of double negative (DN) thymocyte precursors (11). In DN thymocytes, both interleukin-7 (IL-7) and Notch prevent cell death and promote pre-T cell development via activation of glycolytic metabolism and the Akt signaling pathway (12). Following release from the thymus, antigenic stimulation of mature T cells facilitates metabolic changes that support various bioenergetically dependent processes needed for rapid clonal expansion (9). It is proposed that T cells must shift from catabolic to anabolic metabolism in order to rapidly proliferate, likely allowing them to respond to microbial infection [(10) p. 2313]. Indeed, CD8 T cells have the capacity to divide once every 4–6 h (13), a process that is highly energy dependent. Naïve T cells appear to favor energetically efficient processes such as the tricarboxylic acid (TCA) cycle linked to the generation of ATP via oxidative phosphorylation (OXPHOS), which results in roughly 30–32 ATP units per molecule of glucose. In contrast, antigenically stimulated T cells rapidly shift to a dependence on aerobic glycolysis, a less efficient process that yields only two ATP units per molecule of glucose (14, 15). Activated T cells that fail to switch metabolic processes are rendered anergic or undergo apoptosis (16), likely a consequence of failing to accommodate the specific bioenergetic demands of proliferation and differentiation (17, 18). Thus, it is clear that the metabolic status must match the needs of distinct T cell subsets and developmental stages in order for appropriate immune responses to be generated.

With regard to the intracellular signaling involved in metabolic regulation, it has long been appreciated that the mechanistic target of rapamycin (mTOR) has a critical role in T cell activation and metabolism (19). In the context of metabolism, mTOR serves to integrate nutrient and immune signals, including the availability of amino acids and oxygen, as well as the presence of extracellular growth factors. mTOR then acts as an effector to modulate downstream cellular metabolic processes needed to meet the demands of the cell upon stimulation (20). Such processes include protein translational initiation via phosphorylation of S6K1 and eIF4E (21), and lipid biosynthesis through the transcription factor SREBP1 and the nuclear protein receptor PPARγ (22). It is crucial, however, that mTOR does not become prematurely activated until T cells are antigenically stimulated, as the quiescent metabolic state of resting T cells appears to be important for their homeostatic proliferation (23). As detailed later, the energy sensing protein kinase AMPK acts as a master regulator of the metabolic status in resting T cells. Induced by high levels of AMP, AMPK influences mTOR activity through the tumor suppressor tuberous sclerosis complex (24). Comprised of TSC1 and TSC2, the tuberous sclerosis complex itself negatively regulates mTOR activation (25) and is crucial to maintaining homeostatic proliferation of naïve T cells. Supporting this, Yang and colleagues observed that TSC1 deficient naïve T cells possess hyperactive mTOR activity, a resulting loss in quiescence, and a predisposition to undergo apoptosis (26). Though naïve T cells do not initially require mTOR for TCR induced activation, its absence by genetic deletion in mouse CD4 T cells yields a skewed differentiation toward induced T regulatory cells over other effector T cell subsets (27). In addition, mTOR has a central role in the regulation of both activated and long-lived memory T cells as its genetic deletion or pharmacologic inactivation leads to diminished memory T cell activation and function (22, 28, 29).

## Metabolic States in Distinct T Cell Subsets

Naïve T cells utilize efficient oxidative metabolism to maintain their quiescent state. Conversely, once T cells are stimulated by antigen, they must quickly expand their numbers to eliminate an antigenic challenge. This view is reminiscent of the Warburg hypothesis, in which heightened glycolysis observed in cancer cells is thought to allow for rapid tumor cell proliferation, particularly under limiting oxygen tension (30). Alternatively, it has been recently proposed that the switch to aerobic glycolysis is instead necessary to support effector T cell differentiation. Pearce and colleagues demonstrated that blockade of glycolysis prevented the expression of interferon gamma in activated T cells, but did not impair clonal expansion (31). Moreover, the rapid recall response observed in memory T cells, cells that often must respond quite rapidly to antigenic rechallenge, is supported by enhanced respiratory and glycolytic capacity (32, 33). It remains to be determined if this may reflect differential survival of unique subpopulations during clonal expansion. Regardless, distinct metabolic processes are clearly involved in providing for the energetic demands of unique T cell subpopulations, with fatty acid oxidation (FAO) and aerobic glycolysis playing significant roles.

### Fatty acid oxidation

In resting T cells that circulate in the periphery, FAO is the default metabolic state, and the metabolism of these quiescent cells is characterized by a need for basal energy utilization over macromolecular biosynthesis (34). These naïve T cells utilize high energy yielding OXPHOS processes, involving β-oxidation of fatty acids and oxidation of glutamine and pyruvate via the TCA cycle (34). Both naïve CD4 and CD8 T cells also rely on intrinsic IL-7 to maintain homeostasis and quiescent survival (35). Loss of IL-7 receptor (IL-7R) signaling results in defective T cell physiology, characterized by decreased cell size/growth and markedly impaired cell survival (36). The involvement of IL-7R signaling in the maintenance of peripheral T cell homeostasis is complex; it has been recently found that IL-7R signaling must be intermittent and not continuous, as the latter results in sensitization of naïve T cells to cytokine induced cell death (37). While IL-7R signaling promotes survival of quiescent peripheral T cells, and is required for homeostatic proliferation under lymphopenic conditions, it alone is not sufficient to induce naïve T cell activation and the metabolic changes associated with this (38). Instead, with minimal mTOR activity, resting T cells utilize other signaling factors to regulate metabolic pathways. These factors include the nuclear receptors PPARα and PPARγ, both of which regulate fatty acid metabolism and inhibit activation upon TCR engagement (12, 39).

While an increase in glycolysis generally is observed in activated T cells, this is not always the case. Induced regulatory T cells (iT_reg_), differentiated from peripheral CD4^+^ T cells, are a unique subset of CD4^+^ T cells that suppress effector T cells and are vital to immune peripheral tolerance (40). Following a lag phase heavily dependent on glycolysis and glutaminolysis (28), T cells activated in the context of extracellular signals that favor iT_reg_ differentiation (e.g., IL-2 and TGF-β) acquire a metabolic phenotype similar to naïve T cells (41). Relying on lipid oxidation as their primary source of energy, peripherally differentiated iT_regs_ and their thymically derived nT_reg_ counterparts have intermediate to low mTOR activity (18). The distinct metabolic profile of T_reg_ can be replicated through *in vitro* culture with addition of glycolytic or mTOR inhibitors, such as 2-deoxyglucose or rapamycin, respectively (12). As with naïve T cells, PPARα and PPARγ are important for T_regs_, serving as fatty acid sensors, and promoting Foxp3 expression in CD4^+^ T cells activated in the presence of TGF-β (42).

Fatty acid oxidation also plays a vital role in the maintenance of memory T cell pools. Following clearance of an acute viral infection, the antiviral CD8^+^ effector T cell pool is radically depleted, with a loss of 90–95% of virus specific CD8^+^ T cells (43). The surviving cells in turn become long-lived memory T cells (44), possessing unique metabolic characteristics when compared with effector cells (45). Memory CD8^+^ T cells must be able to withstand periods of both antigenic neglect and rapid antigen specific recall through the acquisition of increased spare respiratory capacity (SRC) through biogenesis of mitochondria and increased glycolytic flux (32). Thus, in contrast to their effector counterparts, these long-lived CD8^+^ T cells have significantly enhanced SRC. Memory CD8^+^ T cells share an analogous metabolic profile with resting T cells and T_regs_, primarily engaging in FAO to maintain their survival and homeostasis (46). These metabolic processes are maintained by IL-15 signaling, which facilitates the biogenesis of mitochondria and expression of CPT1A, an enzyme responsible for the rate-limiting step of FAO (32).

### Glycolysis

As noted above, activated T cells switch their metabolic programing to aerobic glycolysis upon antigenic stimulation (15, 47). This may seem counterproductive, as the effective ATP output per glucose molecule taken into the cell is roughly one fifteenth of the units generated via OXPHOS (12). Instead, it has been proposed that this switch is necessary to facilitate the rapid clonal expansion required to eliminate a microbial infection (45). Growth factor stimulation results in enhanced uptake of glucose through the upregulation of the glucose transporter Glut1 on the surface of cells, along with increased expression of the glycolytic enzymes hexokinase and phosphofructokinase (14), processes activated in T cells by TCR ligation (48). Costimulation through CD28 leads to the induction of Akt, thereby enhancing glycolytic activity in T cells (15), and the prevention of growth factor withdrawal induced cell death (17). Supporting a crucial role for Akt in promoting metabolic changes and the survival of activated T cells, ectopic expression of an active form of Akt leads to increased rates of glycolysis and T cell survival, even in the absence of CD28 signaling (49).

**Figure 1:**
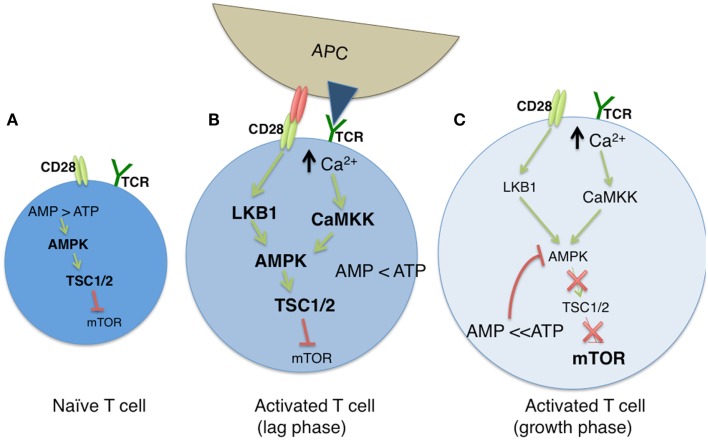
**The modulation of cellular metabolism via T cell receptor (TCR) signaling**. **(A)** In naïve T cells, fatty acid oxidation (FAO) is used to maintain basal cellular metabolism. In this quiescent state, the absence of TCR signaling leads to an elevated AMP to ATP ratio, resulting in sustained AMPK and diminished mTOR activities. **(B)** Upon TCR stimulation by antigen presenting cells (APC), along with CD28-mediated costimulation, glycolysis is greatly enhanced, leading to a diminished AMP:ATP ratio. During a lag phase that precedes T cell clonal expansion, LKB1 and CaMKK (itself induced by high cytosolic Ca^2+^ levels) promote AMPK function despite reduced AMP levels, and mTOR activity remains low via TSC1/2 mediated inhibition. **(C)** As T cells exit the lag phase, heightened levels of ATP block AMPK activity, which results in the loss of TSC1/2 activity. This allows for sustained mTOR function, and subsequent mTOR driven clonal expansion and cell growth.

The AMP-dependent protein kinase AMPK serves a critical regulator of cellular metabolism, both in naïve and newly activated T cells (Figure 1A). In resting cells, a high ratio of AMP to ATP leads to elevated AMPK activity and diminished mTOR function. TCR engagement activates LKB1 and in parallel, increases intracellular Ca^2+^ stores, both leading to an increase in the expression of AMPK (50). LKB1 positively regulates AMPK (51, 52), the latter of which serves as an upstream regulator of TSC1 (52). As TSC1 inhibits mTOR activity in naïve T cells through the tuberous sclerosis complex, AMPK restricts the engagement of metabolic programs associated with clonal expansion. Deletion of the *Tsc1* gene leads to metabolic alterations in T cells, most notably, increases in glucose uptake and glycolytic flux (53). AMPK activity is positively influenced by calcium mobilization via its impact on Ca^2+^ – calmodulin-dependent protein kinase kinase (CaMKK) activity (50, 54). Thus, while AMPK may be associated with a quiescent T cell state, the TCR induced increase of cytosolic Ca^2+^ enhances AMPK activity (Figure 1B). This effectively conserves ATP by inhibiting mTOR associated anabolic processes, and by promoting OXPHOS (50, 55). With time, the resulting increase in the ATP to AMP ratio leads to diminished AMPK activity, and downstream restriction of mTOR is overcome, allowing the T cell to shift its metabolism from FAO to OXPHOS (Figure 1C).

Upon successful engagement of their TCR and the costimulation molecule CD28, the T cell begins to switch toward an activated metabolic program, utilizing aerobic glycolysis, the phosphate pentose pathway (PPP), and glutaminolysis (18). Two transcriptions factors that coordinate metabolic status following T cell activation are Myc and HIF1α (28, 56). Induction of Myc expression in activated T cells is largely responsible for metabolic reprograming at a global gene transcriptome level, as its acute deletion inhibited glycolysis, glutaminolysis, and provoked an overall failure in cell growth and proliferation (28). Myc is also found to intersect with the mTOR pathway, as its loss in activated T cells led to reduced expression and phosphorylation of the downstream mTOR targets S6 and 4E-BP (28). mTOR itself, specifically the mTOR complex 1 (mTORC1), is responsible for activating two key metabolic transcriptional targets, HIF1α and SREBP1/2. Specifically, HIF1α activates downstream targets involved in glycolysis and glucose uptake, while SREBP1/2 activates the PPP and lipid biosynthesis (57). However, while SREBP activity is essential for metabolic reprograming in activated T cells (58), HIF1α appears to play a more selective role in T cell subsets such as Th17 cells (56, 59). It is likely that HIF1β serves a compensatory role in HIF1α-deficient T cells, as loss of HIF1β leads to defective glycolysis, survival, trafficking, and function of CD8^+^ T cells (60).

## Calcium Mobilization Following TCR Ligation

Having considered the modulation of cellular metabolism via TCR proximal signaling pathways, we now turn our attention to calcium mobilization, and how this signaling platform shapes the metabolic response in T cells. As described in detail in this compendium of reviews, TCR stimulation engages numerous signaling cascades, resulting in cellular activation and proliferation. One such molecule that is activated is phospholipase Cγ1 (PLCγ1), which mediates the hydrolysis of phosphatidylinositol-3,4-bisphospahte (PIP_2_) into inositol-1,4,5-trisphosphate (IP_3_) and diacylglycerol (DAG). Ca^2+^ release from ER stores is triggered upon the binding of IP_3_ to inositol trisphosphate receptor (IP_3_R) found on the ER membrane. The two major ion channels that are known to participate in Ca^2+^ release from the ER in response to agonist stimulation are IP_3_Rs (61) and ryanodine receptors (RyRs) (62). Mobilization of intracellular Ca^2+^ triggers store-operated Ca^2+^ entry (SOCE), primarily through calcium release activated Ca^2+^ (CRAC) channels (63). The binding of antigen/MHC to the TCR complex results in the phosphorylation and activation of PLCγ1, which then hydrolyzes phospholipids at the plasma membrane to produce DAG and IP_3_. IP_3_ then binds to its respective receptors in the ER, triggering calcium release into the cytosol. This secondarily activates CRAC channels within the plasma membrane, causing a rapid influx of extracellular calcium that sustains high calcium levels required for T cell activation (64). Upon accumulation, cytosolic calcium binds calmodulin (CaM), inducing a conformational change in CaM that promotes its ability to interact with and activate the protein phosphatase calcineurin (65). Calcineurin dephosphorylates the cytoplasmic subunits (NFATc) of nuclear factor of activated T cells (NFAT) transcription complexes, exposing a nuclear localization sequence that results in their import into the nucleus (66).

Cytosolic calcium is a universal second messenger affecting a variety of cellular processes extending from short- to long-term responses in immune cells (67). Unlike other intracellular messengers, Ca^2+^ is neither synthesized nor metabolized. Rather, Ca^2+^ storage and release are carefully regulated by a series of channels and pumps that maintain precise Ca^2+^ concentrations within distinct cellular compartments. In lymphocytes, activation of immune receptors initiates a signaling cascade culminating in the depletion of intracellular Ca^2+^ stores. Upon depletion, store-operated calcium channels are activated, allowing Ca^2+^ to enter into the cell, primarily through CRAC channels. The increase in intracellular free calcium is essential for lymphocyte activation, since the transcription factor families NFAT, NF-κB, and AP-1 are all targets of Ca^2+^ mediated signaling (68). Remarkably, 75% of all activation-regulated genes in T cells show a dependence on Ca^2+^ influx (68). Supporting this, loss of Ca^2+^ mobilization dramatically reduces, and often prevents, T cell activation and proliferation (68–70). Below, we detail the regulation of intracellular calcium oscillation by CRAC channels and mitochondria, and describe how Ca^2+^ influences the generation of and response to cellular oxidants.

## Store-Operated Ca^2+^ Channels

Calcium release activated calcium channels comprise a widespread and highly conserved Ca^2+^ entry pathway in cells such as lymphocytes, and are activated following the depletion of Ca^2+^ within the ER. This phenomenon is referred to as SOCE and CRAC activity is proportional to the amount of Ca^2+^ depletion within the ER. Over the last decade, the key components of the CRAC channel machinery were identified as STIM1, the ER Ca^2+^ sensor and Orai1/2, pore forming subunits of the CRAC channel (71). Depletion of Ca^2+^ within the ER is detected by the Ca^2+^ sensors STIM1 and STIM2, resulting in the activation of store-operated calcium channels. STIM1 and STIM2 are EF-hand containing single-pass transmembrane proteins with both EF hands capable of binding Ca^2+^. STIM2 is thought to have a lower affinity for Ca^2+^ since STIM1-deficient mouse T cells and fibroblasts bear a severe impairment in store-operated Ca^2+^ influx whereas a deficiency in STIM2 has a less dramatic effect (72).

The luminal EF-hand of STIM1 senses Ca^2+^ depletion within the ER and subsequently causes its oligomerization and translocation toward the plasma membrane (73). STIM1 binds through specialized ER-PM junctions, located within 25 nm of the plasma membrane, to a component of the CRAC channel known as Orai1 (74). Once activated, CRAC channels have a remarkably selective single channel conductance for Ca^2+^, and sensitive Ca^2+^ dependent feedback regulation (75). Association of STIM1 and Orai1 depends on the involvement of STIM1 with the phosphoinositides (PIs) of Orai1. Within STIM1, the cytosolic SOAR/CAD domain contains an alpha-helical domain necessary for Orai1 binding (76, 77). Deletion of the SOAR/CAD domain abolishes store depletion-induced clustering of Orai1 monomers and dramatically reduces SOCE, suggesting this domain is required for the assembly of the SOCE complex at ER-PM junctions (76, 77).

## Mitochondrial Calcium Buffering and Homeostasis

Mitochondria are recognized as essential calcium signaling organelles. Through calcium buffering, mitochondria influence CRAC channel activity (78). With the ability to sense Ca^2+^ microdomains, mitochondria are able to translocate to the immunological synapse upon Ca^2+^ influx, leading to maximal Ca^2+^ uptake. The positioning of mitochondria correlates with the magnitude of local calcium entry via the reduction of Ca^2+^ dependent channel inactivation (79). By co-localizing near ER, mitochondria are better suited to buffer cytosolic Ca^2+^.

Isolated mitochondria take up Ca^2+^ when supported by exogenous electron transport chain (ETC) substrates (80). Although it has been well known that mitochondria are endowed with a complex array of Ca^2+^ transporters, the function of these transporters was not well appreciated until the early 1990s when Pozzan, Rizzuto and colleagues examined aequorin, a Ca^2+^ sensitive bioluminescent protein. They found that agonist-stimulated elevation of cytosolic free Ca^2+^ results in a rapid and transient increase in mitochondrial Ca^2+^, an increase blocked by pretreatment with a mitochondrial uncoupler (81). Prior to this discovery, it was believed that mitochondria failed to release much Ca^2+^ into the cytosol and had little effect on InsP_3_-mediated Ca^2+^ release. It has since become appreciated that mitochondrial matrix Ca^2+^ concentrations are crucial regulators of the Ca^2+^ dependent enzymes of the TCA cycle (82). Mitochondria also play a vital role in the gating of CRAC channels and do so through Ca^2+^ buffering (83).

**Figure 2:**
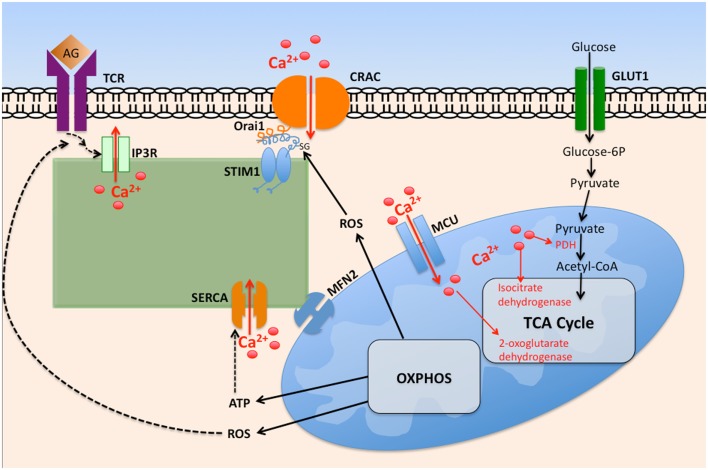
**The interplay between intracellular calcium mobilization and cellular metabolism in T cells**. Ligation of the T cell receptor (TCR) by antigen (Ag) promotes opening of IP_3_R and depletion of ER Ca^2+^ via IP3, itself generated by phospholipase Cγ1. Depletion of ER Ca^2+^ stores promotes STIM1 interaction with Orai1, promoting oligomerization of Orai monomers, thus forming CRAC channels. The resultant increase in cytosolic Ca^2+^ promotes its entry into the mitochondrial matrix via the mitochondrial calcium uniporter (MCU). Elevated matrix Ca^2+^ enhances the activity of key tricarboxylic acid cycle (TCA) enzymes, leading to elevated oxidative phosphorylation (OXPHOS), and ATP generation. Reactive oxygen species (ROS) byproducts of OXPHOS enhance TCR proximal signaling by inhibiting tyrosine phosphatase activity. Elevated cytosolic ROS governs CRAC activity by ROS mediated modification of cysteine residues exposed on Orai1.

Mitochondria act as Ca^2+^ buffers through uptake of Ca^2+^ released by IP_3_R on proximal ER membranes (Figure 2). Mitochondria accomplish this through the ability to detect changes in Ca^2+^ microdomains (84); Ca^2+^ uptake by mitochondria occurs by mitochondrial Ca^2+^ uniporter (MCU) activity across the inner mitochondrial membrane (85). This Ca^2+^ buffering is functionally significant as it modifies the Ca^2+^ dependent inactivation of IP_3_R, ultimately leading to greater Ca^2+^ mobilization. Mitochondria also deplete Ca^2+^ adjacent to the ER, resulting in less available Ca^2+^ for transport into the ER via SERCA pumps. Together, these events result in robust activation of CRAC channels (86, 87). It has been estimated that between 25 and 50% of the Ca^2+^ released from ER is taken up by mitochondria, dependent on cell type, and the Ca^2+^ release channel involved (88). This Ca^2+^ uptake influences mitochondrial metabolic processes, as three rate-limiting enzymes of the Krebs cycle are activated by a rise in Ca^2+^ concentration, subsequently causing an increase in mitochondrial ATP generation (89). These enzymes include pyruvate dehydrogenase (PDH), NAD^+^-isocitrate dehydrogenase (NAD-IDH), and 2-oxoglutarate dehydrogenase (82).

In addition to influencing CRAC channel gating, mitochondria also influence STIM1 trafficking (90). Formation of STIM1 puncta below the plasma membrane is diminished, independently of STIM1 oligomerization, upon strong mitochondrial depolarization. This is accomplished by the mitochondrial protein mitofusin 2 (MFN2), an outer mitochondrial membrane GTPase that couples mitochondrial energetic status to STIM1 migration (90). In MFN2-deficient cells, mitochondrial depolarization fails to influence STIM1 trafficking and Ca^2+^ entry, suggesting that MFN2 confers sensitivity to mitochondrial depolarization. MFN2 assists in tethering mitochondria to the ER and is abundant in the contact sites between the two organelles, though the exact mechanism through which these two organelles interact remains unclear (91). It is speculated that the most likely function of the tethering is to allow mitochondria to localize proximal to Ca^2+^ released from ER. However, the question as to why STIM1 overexpression partially rescues Ca^2+^ entry in MFN2 overexpressing cells has yet to be answered.

While both ER and mitochondria serve as important intracellular reservoirs of Ca^2+^, mitochondria utilize functionally distinct Ca^2+^ transport mechanisms. Mitochondria possess a large electrochemical gradient across their inner membrane (ΔΨ_m_), which acts as a driving force for the uptake of Ca^2+^ (92). Unlike with ER, mitochondrial Ca^2+^ release occurs via ion exchangers, and Ca^2+^ uptake is mediated via a selective ion channel (93). High calcium levels, in combination with other signals, have been known to trigger the opening of channels within the inner mitochondrial membrane, ultimately resulting in cell death (94). The combined action of these mechanisms allows for the rapid cycling of Ca^2+^ across the inner mitochondrial membrane (95).

## Regulation of STIM1/Orai Signaling by Oxidative Stress

Numerous physiological processes, including proliferation, and cell death, are influenced by ROS (96, 97). To prevent oxidative stress, the cell must maintain a balance between superoxide generation, a byproduct of oxidative energy production, and rapid clearance of ROS. The latter is achieved through circulating antioxidants that act as ROS scavengers. Out of the ∼20 types of ROS, hydrogen peroxide (H_2_O_2_) appears to be the major contributor to oxidative damage (96). Of interest here, it has been found that oxidative stress regulates STIM1/Orai1 signaling (98–100). CRAC channels, under the action of STIM1, are stimulated by micromolar concentrations of H_2_O_2_ (98–100). Altered calcium signaling during oxidant stress is attributed to a reduction in the Ca^2+^ binding affinity of STIM1 due to a modification involving its cysteine 56, ultimately resulting in constitutive CRAC activation (100). Upon exposure to ROS, STIM1 becomes S-glutathionylated, thereby triggering its oligomerization and translocation to the plasma membrane (100). Alternatively, Grupe and colleagues demonstrated that H_2_O_2_ enhanced the CRAC current, I_CRAC_-mediated Ca^2+^ influx by activating IP_3_R independent of Orai1 (99). Regardless of the route of entry, both groups demonstrate that an increase an ROS leads to enhanced Ca^2+^ entry into the cell. While several studies support the hypothesis that ROS positively stimulate CRAC channel activity by triggering the oligomerization of STIM1, Bogeski and colleagues report that oxidation via H_2_O_2_ specifically blocks the activation of Orai1 channels in human T helper lymphocytes (98). It is speculated that the oxidation of the cysteine, found within the extracellular loop of Orai1, may lock the channel in the closed conformation. Nevertheless, cells with an SOCE deficiency are more susceptible to oxidative stress. Despite having an up-regulated basal antioxidant response, STIM1-deficient mouse embryonic fibroblasts experience an imbalance of ROS production and detoxification upon addition of exogenous oxidants (101). Henke and colleagues conclude that functional SOCE machinery is required to balance ROS production and the cellular antioxidant defense system. Collectively, these studies support the notion that the cellular oxidative stress response is influenced through a dynamic interplay between ROS balance and Ca^2+^ influx.

## Oxidant Dependence of T Cell Activation

Oxidative stress is provoked by an imbalance between the production of mitochondrial superoxide and insufficient scavenging of ROS, ultimately resulting in a wide range of pathological conditions (102). The dynamics of ROS production and scavenging can be detected using several fluorescent dyes, including reduced forms of ethidium bromide (DHE) (103), fluorescein (DCFDA) (104, 105), rhodamine (DHR) (106), and hydroethidine (HE) (107). These dyes remain relatively non-fluorescent until they are oxidized by ROS, and their unquenched fluorescence correlates with increased oxidation. These intracellular dyes provide sensitive probes for multiple reactive species and allow for the detection of fluorescent products using fluorimetry and flow cytometry. In T cells, DCFDA, DHR, and DHE have been utilized to examine ROS production from a variety of stimuli including mitogens (108), viral and bacterial superantigen (109, 110), and TCR peptide agonists (111, 112). ROS generation has been observed following stimulation of T cells by other ligands including TGF-β, insulin, angiotensin II, and EGF (113–116). Collectively, these studies suggest that ROS function as secondary messengers necessary for complete T cell activation. Superoxide is generated from the mitochondrial ETC through complexes I, II, and III (117). Complexes I and II emit superoxide into the mitochondrial matrix, upon which superoxide dismutase 2 (SOD2) converts it into hydrogen peroxide (H_2_O_2_). In contrast, complex III emits superoxide into both the matrix and intermembrane spaces. Intermembrane-space superoxide is capable of reaching the cytosol through voltage-dependent anion channels (VDAC) without conversion into H_2_O_2_ (118, 119).

Recently, Sena et al. reported that blocking mitochondrial ROS, specifically generated at complex III, results in the inhibition of CD3/CD28 induced IL-2 expression. Additionally, extracellular calcium influx and subsequent Ca^2+^ uptake by mitochondria were both required for mitochondrial ROS production (120). These findings support the hypothesis that complex III produced mitochondrial ROS, a byproduct of mitochondrial metabolism, facilitate T cell activation and functionality. However, the mechanisms through which oxidants may regulate T cell responses upon stimulation remain poorly defined. Importantly, the timing and subcellular localization of ROS generation are likely of greater influence in T cell responses than overall redox balance (102, 121, 122). ROS clearly play key roles in modulating T cell activation and differentiation, but vital details regarding the influence of these oxidants on specific signaling molecules remain to be clarified.

## DRAK2 as a an Intermediary in Calcium and Metabolic Signaling in T Cells

One molecule expressed in T cells that may potentially link calcium and ROS signaling to cellular metabolic regulation is DRAK2/STK17B. DRAK2 is a serine/threonine kinase of the death associated protein kinase (DAPK) family (123), which is comprised of five known members (DAPK1, DAPK2, DAPK3, DRAK1, and DRAK2). All of these kinases are capable of inducing cell death when ectopically expressed in cells, and the prototype DAPK1 possesses a calmodulin regulatory domain that, through binding to calmodulin/Ca^2+^, regulates its catalytic activity (124). While DRAK2 lacks a calmodulin binding domain, like other DAPK family members, DRAK2 has been shown to induce apoptosis upon overexpression in cell lines (125), and its phosphorylation and nuclear translocation participates in ultraviolet light induced cell death (126).

Although broadly expressed at low levels (127), DRAK2 expression is enriched in cells of hematopoietic origin (128). Loss of expression by virtue of a germline deletion of the *Drak2* gene leads to hyperactive Ca^2+^ mobilization in T cells, especially under suboptimal TCR stimulus conditions (128, 129), supporting the hypothesis that DRAK2 acts as a negative regulator of TCR signaling (130). The notion that DRAK2 serves as a rheostat in calcium signaling following TCR signaling is supported by the finding that it is itself activated by calcium mobilization, and that its ectopic expression in double positive thymocytes raises the threshold for both negative and positive selection (131, 132). Enigmatically, loss of the *Drak2* gene leads to diminished autoimmune susceptibility, with reduced clinical severity observed in animal autoimmune models for multiple sclerosis (experimental autoimmune encephalomyelitis) and type I diabetes-prone non-obese diabetic (NOD) mice (129, 133–135). The reduced EAE susceptibility is due, at least in part, to diminished survival of activated effector T cells, perhaps a consequence of impaired calcium homeostasis (133–135).

Using a mass spectrometry based approach, we determined that DRAK2 possesses several autophosphorylation sites, most notably Ser10, Ser12, and Ser351. Using antibodies specific for phosphorylation at Ser12, it was found that TCR stimulation induces DRAK2 Ser12 autophosphorylation (131), and this requires entry of extracellular Ca^2+^ into T cells. The SERCA inhibitor thapsigargin was found to potently induce DRAK2 autophosphorylation on Ser12, supporting the hypothesis that ER store-operated calcium entry modulates DRAK2 catalytic activity. Recent studies have revealed that TCR induced DRAK2 activation is highly dependent on CRAC function, as DRAK2 was poorly activated following antigenic stimulation in Orai2-deficient T cells (136). Using a panel of small molecule inhibitors and RNA interference approaches, it was found that DRAK2 activation by antigenic stimulation requires protein kinase D (PKD).

**Figure 3:**
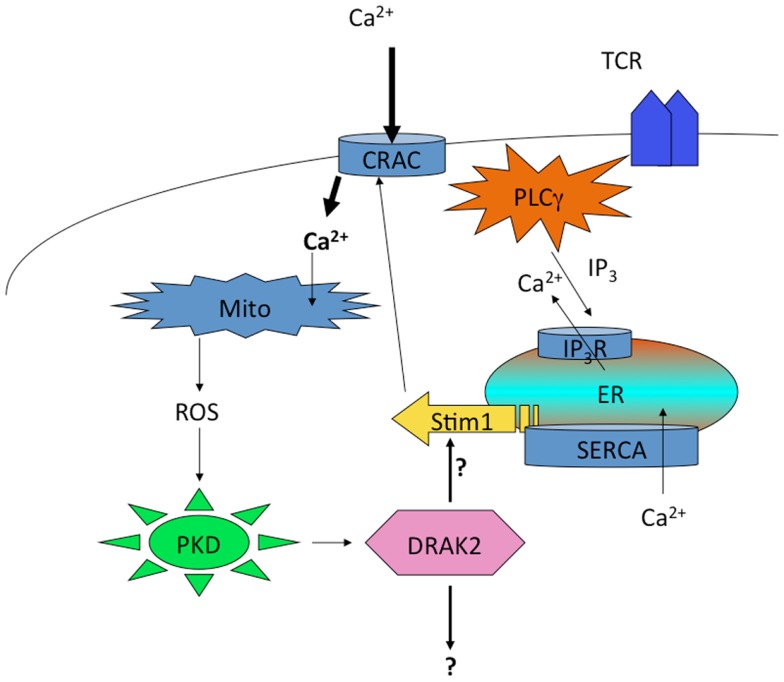
**Regulation of DRAK2 activity in T cells via Ca^2+^-induced respiratory burst**. TCR induced Ca^2+^ depletion from ER stores results in opening of CRAC channels. This high cytosolic [Ca^2+^] promotes the induction of TCA cycle and OXPHOS in mitochondria, the latter of which release ROS as an OXPHOS byproduct. In addition to inhibiting phosphatases, ROS activate PKD, which itself binds to and activates DRAK2 (likely through transphosphorylation on DRAK2). DRAK2 then impacts Ca^2+^ signaling by altering SERCA activity (hypothetical), or other unknown substrates.

Curiously, DRAK2 catalytic activity in activated T cells also depends on mitochondrial ROS, as antioxidants blocked Ser12 autophosphorylation (136). Since the electron transport complex III inhibitor FCCP led to Ser12 phosphorylation independent of antigenic stimulation of T cells, these findings demonstrate that ROS are both necessary and sufficient to promote DRAK2 catalytic activity. PKD activity is itself subject to activation by mitochondrial ROS (137, 138), and thus ROS induced by enhanced mitochondrial respiration that results from TCR stimulation likely participates in PKD-mediated DRAK2 activation (Figure 3). The specific nature of the interaction between mitochondrial ROS and DRAK2’s substrates is poorly understood. However, as DRAK2-deficient T cells bear enhanced CRAC activity (136), and DRAK2-transgenic thymocytes have diminished Ca^2+^ mobilization following TCR stimulation (132), it is likely that DRAK2 may phosphorylate an intermediate in CRAC signaling. DRAK2 has been shown to phosphorylate S6K1 (139), an important target of mTOR and of TCR signaling that impacts cellular metabolism (22, 57, 140). Thus, DRAK2 may itself serve as an important link between calcium and mTOR signaling, impacting the differentiation and survival of T cell subsets selectively required for immune responsiveness.

## Conclusion

Research into the regulation of CRAC activity, and the unique involvement of metabolic signaling in T cells, has been intense over the last several years. It is becoming quite evident that both signaling paradigms are highly regulated and vital to T cell activation, differentiation, and homeostasis. These pathways also appear to play selective roles in distinct T cell subsets, with the potential that manipulation of such pathways could influence the balance between immunity vs. self-tolerance in different disease states. A significant challenge to immunologists is to develop selective therapies to influence this balance. In the case of autoimmune disorders, the difficulty is to block autoreactive T cell function without promoting general immunosuppression. In the case of chronic microbial pathogen infections and cancer, therapeutic approaches to reinvigorate antimicrobial or anti-tumor T cell responsiveness are highly sought after. It is thus imperative that novel methods are considered to influence the appropriate immune response. Given the unique crosstalk between calcium mobilization and bioenergetic metabolism, there may be an opportunity to uniquely affect the outcome of immune therapies by targeting the molecules that mediate this crosstalk.

## Conflict of Interest Statement

The authors declare that the research was conducted in the absence of any commercial or financial relationships that could be construed as a potential conflict of interest.
